# Associations of MIND and DI-GM dietary scores with depression, anxiety, and gut microbiota in patients with colon cancer: a cross-sectional study

**DOI:** 10.3389/fnut.2025.1655051

**Published:** 2025-08-25

**Authors:** Yaqin Meng, Jing Tian, Xiu Xiu Li, Zhou Xu

**Affiliations:** 1 Department of Psychiatry, The First Hospital of Shanxi Medical University, Taiyuan, Shanxi, China; 2 Department of Gastroenterology, Shanxi Province Cancer Hospital/Shanxi Hospital Affiliated to Cancer Hospital, Chinese Academy of Medical Sciences/Cancer Hospital Affiliated to Shanxi Medical University, Taiyuan, Shanxi, China

**Keywords:** diet, Mediterranean, biomarkers, depression, gut microbiota, colon neoplasms

## Abstract

**Background:**

Dietary patterns influence psychological health, systemic inflammation, and gut microbiota composition in colon cancer patients. This study evaluates the associations of the Mediterranean-DASH Intervention for Neurodegenerative Delay (MIND) score and the Dietary Index for Gut Microbiota (DI-GM) with psychological outcomes, inflammatory markers, gut microbiota diversity (Shannon index) and composition (Firmicutes/Bacteroidetes ratio), and tumor biomarkers in colon cancer patients.

**Methods:**

A cross-sectional study was conducted on 630 colon Cancer patients. Multivariate linear regression models adjusted for demographic, clinical, and dietary factors assessed associations of MIND and DI-GM scores with depression, anxiety (HADS), sleep quality (PSQI), quality of life (FACT-C), inflammatory markers (CRP, IL-6, fecal calprotectin), F/B ratio, and tumor biomarkers (CEA, CA19-9).

**Results:**

Higher MIND and DI-GM scores were significantly associated with better psychological outcomes and reduced systemic inflammation. Each one-unit increase in the MIND score was associated with lower depression (*β* = −1.16, 95% CI: −2.24 to −0.08) and anxiety (β = −2.48, 95% CI: −4.01 to −0.95). Similarly, DI-GM was inversely associated with depression (β = −1.36, 95% CI: −1.53 to −1.20), anxiety, and inflammatory markers. Tumor biomarkers such as CA19-9 and CEA showed significant inverse associations with both scores, especially DI-GM (CA19-9: *β* = −3.11, 95% CI: −4.93 to −1.29; CEA: β = −0.38, 95% CI: −0.55 to −0.20). The F/B ratio partially mediated the relationship between dietary scores and psychological outcomes but not inflammatory markers.

**Conclusion:**

Adherence to MIND and DI-GM dietary patterns is associated with better psychological outcomes, lower inflammation, and favorable gut microbiota in colon cancer patients. DI-GM may better capture diet–gut microbiota–inflammation links, highlighting diet as a target to improve patient well-being.

## Introduction

Colon cancer is a major global health concern, ranking among the most common cancers worldwide and contributing significantly to cancer-related morbidity and mortality (1). Beyond its physical burden, colon cancer is frequently accompanied by psychological distress, including depression and anxiety, which adversely affect treatment adherence, quality of life, and clinical outcomes (2). As the understanding of cancer pathophysiology deepens, increasing attention has been directed toward modifiable factors such as diet and gut microbiota, which may influence both tumor progression and mental health (3, 4).

The gut–brain axis—a bidirectional communication network linking the gastrointestinal tract and central nervous system—is recognized as a critical pathway through which dietary and microbial factors impact psychological well-being (5). Diet plays a central role in shaping gut microbial composition and function, which in turn affects neuroinflammation, neurotransmitter metabolism, and mood regulation (6, 7). This triad of diet, microbiota, and mental health has gained considerable attention, particularly in the context of chronic diseases, including cancer.

The Mediterranean-DASH Intervention for Neurodegenerative Delay (MIND) diet, originally designed to delay cognitive decline, has been associated with reduced risk of depression and anxiety in various non-cancer populations (8–10). In parallel, the Dietary Index for Gut Microbiota (DI-GM), a novel dietary score reflecting the microbiota-modulating potential of diet (11), has been proposed as a tool to assess the gut health impact of dietary patterns (12). Both indices provide biologically plausible frameworks to explore the impact of nutrition on mental health through microbial pathways (13).

Although there is growing interest in the links between diet, gut microbiota, and mental health in colon cancer patients, few studies have examined these relationships comprehensively. Most research focuses on general diets or single nutrients, overlooking microbiota-driven dietary indices and their impact on psychological symptoms (14, 15). For instance, the Dietary Inflammatory Index (DII) has been used to study inflammation and depression in colon cancer, showing higher DII scores relate to increased inflammation, worse quality of life, and psychological distress (10, 16). However, these studies rarely assess gut microbiota or the diet–microbiota–brain connection, often examining these factors separately, which limits understanding of how diet influences mental health through microbial pathways in this population. This study addresses this critical gap by investigating the associations between MIND and DI-GM dietary scores, symptoms of depression and anxiety, and gut microbiota profiles in patients with colon cancer. By evaluating these interrelated domains concurrently, we aim to elucidate the potential role of diet and microbiota in shaping psychological health in this population.

To our knowledge, this is the first cross-sectional study to explore the integrated relationship between MIND and DI-GM dietary patterns, psychological distress, and gut microbiota composition (Shannon index and Firmicutes-to-Bacteroidetes ratio) in colon cancer patients. These findings may provide a foundation for developing microbiota-targeted dietary strategies to support mental health and improve quality of life in cancer care.

## Methods

### Study design and setting

This cross-sectional observational study was conducted at Shanxi Province Cancer Hospital, China, from January 2024 to December 2024. The primary objective was to evaluate the relationships among dietary patterns, gut microbiota composition, digestive enzyme activities, and psychological health in adults diagnosed with colon cancer. The research protocol adhered strictly to the STROBE guidelines for reporting observational studies.

### Participants

**Figure 1:**
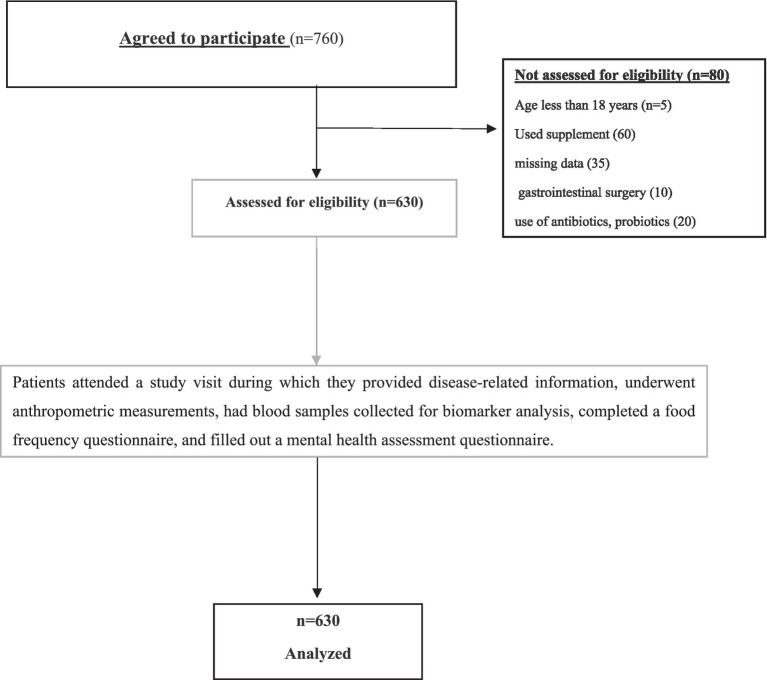
Flowchart of study participants’ enrollment and inclusion.

A total of 630 adult patients (≥18 years) with histologically confirmed colon cancer (all stages I through IV) were consecutively recruited from the hospital’s oncology outpatient clinics and inpatient wards. Eligibility criteria included: confirmed diagnosis of colon cancer, ability to provide informed consent, and capability to complete dietary and psychological assessments. Exclusion criteria comprised: recent use of antibiotics, prebiotic, synbiotic, and probiotics, or immunosuppressants within 4 weeks prior to sampling; diagnosed psychiatric disorders predating cancer diagnosis; recent major gastrointestinal surgery other than temporary or permanent stoma placement; or inability to provide stool samples. Participants with severe cognitive impairments or acute infections at the time of recruitment were also excluded to reduce confounding factors affecting psychological or microbiota assessments. A detailed flowchart illustrating participant recruitment, eligibility screening, enrollment, exclusions, and final sample analyzed is provided (Figure 1).

### Ethical considerations

The study protocol received approval from the Medical Research Ethics Committee of the First Hospital of Shanxi Medical University (Approval No. 2024-506). Written informed consent was obtained from all participants before enrollment. Confidentiality and privacy were ensured according to the Declaration of Helsinki.

### Sample size calculation

The sample size was calculated using G*Power 3.1 based on the primary objective of assessing associations between dietary scores (MIND and DI-GM) and psychological outcomes (depression and anxiety). Assuming a medium effect size (Cohen’s *f^2^* = 0.15), power of 90%, alpha of 0.05, and inclusion of up to 15 covariates in the regression model, a minimum of 490 participants was required (17). To account for an estimated 20% non-response or incomplete data, 630 participants were recruited.

### Data collection procedures

#### Sociodemographic and clinical data

Trained research staff collected detailed sociodemographic information including age, sex, body mass index (BMI), smoking status, physical activity level (self-reported), and educational background through structured interviews. Clinical information was abstracted from medical records, including tumor stage (I–IV, according to AJCC TNM classification), presence and type of stoma (none, temporary, permanent), chemotherapy status (yes/no), radiation therapy status (yes/no), presence of metastasis (yes/no), and other relevant comorbidities. Data quality was ensured by double-entry and cross-verification of clinical records. All questionnaires were administered by trained personnel to reduce response bias, and participants were given standardized instructions to improve reliability.

### Dietary assessment

Participants completed a validated semi-quantitative Food Frequency Questionnaire (FFQ) designed to capture usual dietary intake over the preceding 12 months (18). The FFQ was administered immediately after diagnosis, capturing dietary intake over the preceding 12 months. This recall period may have included dietary changes made in response to diagnosis or treatment planning. Two dietary indices were calculated from the FFQ data to quantify adherence to healthful dietary patterns. The FFQ included 149 food items, categorized into food groups consistent with Mediterranean and microbiota-supportive dietary patterns. Using FFQ data, two dietary indices were calculated:

#### MIND diet score

Derived based on the consumption frequency of 15 components that combine elements of the Mediterranean and DASH diets, emphasizing intake of green leafy vegetables, berries, nuts, whole grains, fish, poultry, olive oil, and limiting red meats, butter, cheese, pastries, and fried foods. The total score ranged from 0 to 15, with higher scores indicating greater adherence (19).

#### Dietary index for gut microbiota

A composite score capturing intake of dietary components known to enhance gut microbial diversity and function, including dietary fiber, fermented foods (e.g., yogurt, kimchi), polyphenol-rich fruits and vegetables, and whole grains. The dietary index for gut microbiota (DI-GM) was computed on a continuous scale based on weighted consumption frequencies (11).

### Psychological assessments

Participants’ psychological status was evaluated using a set of standardized and validated instruments to assess depressive and anxiety symptoms. The Hospital Anxiety and Depression Scale (HADS) was administered, comprising two subscales: HADS-Anxiety (HADS-A) and HADS-Depression (HADS-D), each with 7 items scored from 0 to 3, yielding subscale scores between 0 and 21. Scores of ≥8 on either subscale were considered indicative of clinically relevant anxiety or depression (20).

### Quality of life and sleep assessment

The Functional Assessment of Cancer Therapy–Colon (FACT-C), a validated colon cancer–specific questionnaire, was employed to evaluate physical, social/family, emotional, and functional well-being, alongside additional concerns specific to colon cancer. Scores range from 0 to 136, with higher scores reflecting better quality of life (21).

Sleep quality was assessed using the Pittsburgh Sleep Quality Index (PSQI), a 19-item questionnaire that yields a global score between 0 and 21. A PSQI global score > 5 was defined as indicative of poor sleep quality (22).

### Gut microbiota composition analysis

Stool samples were collected by participants using sterile containers and transported in temperature-controlled conditions within 24 h prior to the clinical visit. Upon receipt, samples were aliquoted and stored at −80°C. Microbial DNA was extracted using the QIAamp DNA Stool Mini Kit (Qiagen) following optimized protocols, including mechanical disruption for effective bacterial lysis. Gut microbiota composition was conducted by amplifying the V3–V4 regions of the 16S rRNA gene and sequencing on the Illumina MiSeq platform (2 × 250 bp). Alpha diversity was assessed using the Shannon index, and the Firmicutes-to-Bacteroidetes (F/B) ratio was calculated from relative abundances to reflect microbial balance (23).

### Fecal biomarker assays

Activities of digestive enzymes—amylase, and lipase—were measured using commercially available colorimetric assay kits (e.g., Sigma-Aldrich), which quantify enzyme activity based on substrate hydrolysis producing chromogenic products. Enzyme activities were normalized to fecal weight (per gram of stool). Intestinal inflammation was assessed by measuring fecal calprotectin levels via enzyme-linked immunosorbent assay (ELISA), utilizing kits with established specificity and sensitivity. All assays were conducted in duplicate with inclusion of appropriate controls to ensure assay reliability.

### Statistical analysis

All statistical analyses were conducted using Stata version 18.0. Continuous variables were summarized as means ± standard deviations or medians with interquartile ranges, depending on normality assessed by the Shapiro–Wilk test, while categorical variables were presented as frequencies and percentages. Due to the large number of statistical tests performed, the risk of Type I errors (false positives) is increased; however, no correction for multiple testing was applied given the exploratory nature of this study. Group differences across tertiles of MIND and DI-GM scores in psychological symptoms, gut microbiota diversity, and digestive enzyme activity were tested using one-way ANOVA or Kruskal-Wallis tests, as appropriate. Multivariate linear regression models were used to assess associations between dietary scores and psychological outcomes (HADS-D, HADS-A), adjusting for relevant covariates. Mediation analyses were performed to determine whether the gut microbiota F/B ratio mediated the associations between dietary scores and psychological symptoms. Indirect effects with 95% bias-corrected confidence intervals excluding zero were considered statistically significant. A two-tailed *p*-value < 0.05 indicated statistical significance. Tumor marker analyses were conducted across all stages combined; subgroup analyses by stage were not performed due to limited sample size within certain tertiles.

## Results

### Baseline characteristics by MIND score tertiles

The study cohort included 630 colon cancer patients divided into MIND score Tertiles (T1–T3). The study population had a mean age of 57.41 ± 4.73 years, with 57% male participants, a mean BMI of 24.92 ± 1.28 kg/m^2^, and a tumor stage distribution of approximately 19.7% stage I, 36.3% stage II, 31.7% stage III, and 12.2% stage IV. Mean MIND scores differed significantly across Tertiles (*p* < 0.001), increasing from 9.15 ± 0.38 in T1 to 13.15 ± 0.40 in T3. Age did not differ significantly (*p* = 0.259), but BMI was lower in T2 and T3 compared to T1 (*p* < 0.001). The DI-GM score increased alongside MIND Tertiles, peaking at 11.30 ± 0.38 in T3 (*p* < 0.001). No significant differences were observed across tertiles in sex distribution (*p* = 0.230), tumor stage (*p* = 0.272), or receipt of chemotherapy (*p* = 0.841).

Higher MIND scores were associated with lower depression (HADS-D), anxiety (HADS-A), and better sleep quality (PSQI) (all *p* < 0.001). Higher MIND scores were also associated with better quality of life (FACT-C) (*p* < 0.001).

Biomarker analysis revealed higher BDNF levels and increased microbial diversity (Shannon index) with increasing MIND scores (*p* < 0.001), while the F/B ratio declined significantly (*p* < 0.001). Digestive enzyme activities (amylase, lipase) and inflammatory markers (calprotectin, IL-6, CRP) differed significantly across Tertiles (*p* < 0.001). Higher MIND scores were associated with higher fecal butyrate concentrations (p < 0.001). Hemoglobin, WBC, neutrophil-to-lymphocyte ratio (NLR), platelets, and renal function markers showed no significant differences. However, albumin and liver enzymes (AST, ALT) differed (*p* < 0.05). Tumor markers CEA and CA19-9 were significantly lower in participants with higher MIND scores (CEA: *p* = 0.002; CA19-9: *p* = 0.016). Dietary fiber intake was higher in higher MIND groups (*p* < 0.001), whereas total energy and macronutrient intake remained stable. Blood pressure, glucose, and lipid profiles did not differ significantly (Table 1).

**Table 1:** Baseline demographic, clinical, and biomarker characteristics by MIND score tertiles.

Variables	Total (*N* = 630)	T1 (Low MIND, *N* = 215)	T2 (Moderate MIND, *N* = 206)	T3 (High MIND, *N* = 209)	*p*-value
MIND (score)	11.27 ± 1.74	9.15 ± 0.38	11.56 ± 0.86	13.15 ± 0.40	<0.001
Sex, male, *n* (%)	359 (57)	113 (18)	125 (19.8)	121 (19.2)	0.230
Tumor stage, *n* (%)					
Stage I	124 (19.7)	40 (6.3)	43 (6.8)	41 (6.5)	0.272
Stage II	229 (36.3)	74 (11.7)	79 (12.5)	76 (12.1)	
Stage III	200 (31.7)	76 (12.1)	66 (10.5)	58 (9.2)	
Stage IV	77 (12.2)	25 (4)	18 (2.9)	34 (5.4)	
Chemotherapy, *n* (%)	469 (74.4)	160 (25.4)	156 (24.8)	153 (24.3)	0.841
Age (years)	57.41 ± 4.73	57.81 ± 4.60	57.34 ± 5.25	57.07 ± 4.55	0.259
BMI (kg/m^2^)	24.92 ± 1.28	25.47 ± 1.24	24.76 ± 1.42	24.53 ± 0.97	<0.001
DI-GM (score)	9.66 ± 1.59	7.72 ± 0.42	10.04 ± 0.79	11.30 ± 0.38	<0.001
HADS-D (score)	5.17 ± 2.35	7.88 ± 1.30	4.57 ± 1.4	2.96 ± 0.45	<0.001
HADS-A (score)	5.97 ± 2.35	8.68 ± 1.3	5.37 ± 1.4	3.76 ± 0.45	<0.001
PSQI (score)	5.25 ± 1.60	6.96 ± 0.37	5.21 ± 1.21	3.52 ± 0.34	<0.001
FACT-C (score)	78.31 ± 10.14	66.62 ± 3.64	80.1 ± 6.75	88.54 ± 2.15	<0.001
BDNF (ng/mL)	17.18 ± 7.4	15.60 ± 7.39	16.81 ± 7.07	19.18 ± 7.33	<0.001
Shannon (score)	3.74 ± 0.53	3.08 ± 0.19	3.92 ± 0.29	4.24 ± 0.12	<0.001
F/B (ratio)	2.09 ± 0.61	2.8 ± 0.27	1.96 ± 0.34	1.48 ± 0.16	<0.001
Amylase (U/L)	86.27 ± 3.86	87.90 ± 3.74	85.77 ± 4.26	85.1 ± 2.92	<0.001
Lipase (U/L)	74.77 ± 3.86	76.4 ± 3.74	74.27 ± 4.26	73.6 ± 2.92	<0.001
Calprotectin (μg/g)	57.98 ± 19.42	78.9 ± 10.57	55.17 ± 14.49	39.26 ± 3.13	<0.001
IL-6 (pg/mL)	5.17 ± 1.74	7.29 ± 0.61	4.71 ± 0.91	3.46 ± 0.47	<0.001
CRP (mg/L)	3.41 ± 1.33	4.98 ± 0.67	3.05 ± 0.72	2.12 ± 0.3	<0.001
Butyrate (mmol/L)	6.49 ± 2.52	3.58 ± 0.73	6.68 ± 1.06	9.35 ± 0.93	<0.001
WeightLoss6mo (kg)	2.77 ± 3.13	3.02 ± 3.25	3.17 ± 3.31	2.14 ± 2.72	0.025
Hb (g/dL)	12.00 ± 1.36	11.9 ± 1.4	12.01 ± 1.42	12.08 ± 1.24	0.420
WBC (10^9^/L)	7.19 ± 1.94	7.33 ± 1.96	7.22 ± 2.09	7.01 ± 1.76	0.250
NLR (ratio)	2.30 ± 0.93	2.31 ± 0.95	2.28 ± 1.05	2.3 ± 0.84	0.948
Platelets (10^9^/L)	229.97 ± 51.86	231.9 ± 53.7	230.52 ± 55.95	227.51 ± 46.28	0.676
Albumin (g/dL)	3.82 ± 0.48	3.78 ± 0.51	3.78 ± 0.49	3.89 ± 0.43	0.039
AST (U/L)	33.16 ± 13.76	34.71 ± 14.21	34.15 ± 14.75	30.65 ± 11.9	0.005
ALT (U/L)	30.32 ± 12.66	31.81 ± 13.11	31.29 ± 13.53	27.89 ± 10.93	0.003
Creatinine (mg/dL)	1.06 ± 0.29	1.07 ± 0.3	1.06 ± 0.31	1.05 ± 0.25	0.811
BUN (mg/dL)	17.73 ± 4.68	17.88 ± 4.84	17.86 ± 5.06	17.46 ± 4.11	0.593
CEA (ng/mL)	4.49 ± 3.09	4.89 ± 3.22	4.17 ± 3.3	3.87 ± 2.64	0.002
CA19-9 (U/mL)	31.57 ± 23.99	35.1 ± 25.18	31.2 ± 26.09	28.39 ± 19.97	0.016
Energy (kcal)	1946.15 ± 242.81	1950.6 ± 249.2	1942.43 ± 241.58	1944.86 ± 238.4	0.939
Fiber (g)	18.52 ± 7.4	16.94 ± 7.39	18.15 ± 7.07	20.52 ± 7.33	<0.001
Carbs (g)	274.49 ± 19.54	275.23 ± 20.20	274.20 ± 19.54	274.02 ± 18.93	0.789
Protein (g)	61.49 ± 13.52	61.72 ± 13.77	61.49 ± 13.58	61.26 ± 13.26	0.940
Fat (g)	66.90 ± 13.75	66.98 ± 14.1	66.63 ± 13.6	67.08 ± 13.59	0.942
Systolic (mmHg)	128.12 ± 8.23	128.31 ± 8.3	128.27 ± 8.24	127.76 ± 8.17	0.747
Diastolic (mmHg)	81.85 ± 4.11	81.91 ± 4.32	81.78 ± 3.96	81.85 ± 4.06	0.944
Glucose (mg/dL)	91.24 ± 4.14	91.3 ± 4.42	91.33 ± 4.02	91.09 ± 3.97	0.811
HDL (mg/dL)	45.36 ± 6.97	45.3 ± 7.03	45.49 ± 6.85	45.29 ± 7.05	0.950
TG (mg/dL)	129.91 ± 4.88	129.81 ± 5.08	130.00 ± 4.62	129.92 ± 4.96	0.926
LDL (mg/dL)	95.07 ± 6.68	95.15 ± 6.73	95.05 ± 6.38	91	0.973
Cholesterol (mg/dL)	186.46 ± 11.69	186.57 ± 11.89	186.2 ± 11.27	186.6 ± 11.92	0.929

Data are presented as mean ± standard deviation. *p*-values were calculated using ANOVA or Kruskal-Wallis tests where appropriate. A *p*-value < 0.05 was considered statistically significant. MIND, Mediterranean-DASH Intervention for Neurodegenerative Delay; DI-GM, Dietary Inflammatory Index—Gut Microbiota; HADS-D, Hospital Anxiety and Depression Scale—Depression Subscale; HADS-A, Hospital Anxiety and Depression Scale—Anxiety Subscale; PSQI, Pittsburgh Sleep Quality Index; FACT-C, Functional Assessment of Cancer Therapy—colon cancer; BDNF, Brain-Derived Neurotrophic Factor; F/B ratio, Firmicutes to Bacteroidetes ratio; IL-6, Interleukin-6; CRP, C-reactive protein; WBC, White Blood Cell count; NLR, Neutrophil-to-Lymphocyte Ratio; AST/ALT, Aspartate Aminotransferase/Alanine Aminotransferase; BUN, Blood Urea Nitrogen; CEA, Carcinoembryonic Antigen; CA19-9, Cancer Antigen 19-9; HDL/LDL/TG, High-Density Lipoprotein/Low-Density Lipoprotein/Triglycerides.

### Baseline characteristics by DI-GM score tertiles

When stratified by DI-GM Tertiles, mean DI-GM scores ranged from 7.70 ± 0.39 in T1 to 11.36 ± 0.30 in T3 (*p* < 0.001). Age was comparable across groups (*p* = 0.334), but BMI was significantly lower in higher DI-GM Tertiles (*p* < 0.001). MIND scores rose significantly with increasing DI-GM Tertiles (*p* < 0.001). Psychological measures mirrored the trends seen with MIND scores, showing better depression, anxiety, sleep quality, and quality of life (all *p* < 0.001). Biomarkers including BDNF, Shannon index, and F/B ratio better significantly with higher DI-GM adherence (*p* < 0.001). Amylase and lipase activities varied significantly across Tertiles (*p* < 0.001). Inflammatory markers decreased with higher DI-GM scores (*p* < 0.001). Fecal butyrate was elevated in the highest quartile (*p* < 0.001). Hemoglobin, and NLR showed significant differences across DI-GM Tertiles (*p* = 0.045, 0.022, and 0.021, respectively). Other clinical laboratory values and tumor markers did not differ substantially (Table 2).

**Table 2:** Baseline demographic, clinical, and biomarker characteristics by DI-GM score tertiles.

Variables	Total (*N* = 630)	T1 (Low DI-GM, *N* = 210)	T2 (Moderate DI-GM, *N* = 210)	T3 (High DI-GM, *N* = 210)	*p*-value
DI-GM (score)	9.66 ± 1.59	7.70 ± 0.39	9.94 ± 0.75	11.36 ± 0.30	<0.001
MIND (score)	11.27 ± 1.74	9.13 ± 0.37	11.55 ± 0.89	13.11 ± 0.39	<0.001
Age (years)	57.41 ± 4.73	57.79 ± 4.65	57.11 ± 4.99	57.33 ± 4.53	0.334
BMI (kg/m^2^)	24.92 ± 1.28	25.45 ± 1.25	24.64 ± 1.49	24.68 ± 0.88	<0.001
Tumor stage, *n* (%)					0.272
Stage I	124 (19.7)	40 (6.3)	31 (4.9)	53 (8.4)	
Stage II	229 (36.3)	74 (11.7)	82 (13.0)	73 (11.6)	
Stage III	200 (31.7)	71 (11.3)	71 (11.3)	58 (9.2)	
Stage IV	77 (12.2)	25 (4.0)	26 (4.1)	26 (4.1)	
HADS-D (score)	5.17 ± 2.35	7.90 ± 1.3	4.60 ± 1.45	2.99 ± 0.48	<0.001
HADS-A (score)	5.97 ± 2.35	8.70 ± 1.29	5.40 ± 1.44	3.79 ± 0.47	<0.001
PSQI (score)	5.25 ± 1.60	6.96 ± 0.38	5.24 ± 1.24	3.54 ± 0.36	<0.001
FACT-C (score)	78.31 ± 10.14	66.55 ± 3.65	79.89 ± 6.95	88.46 ± 2.16	<0.001
BDNF (ng/mL)	17.18 ± 7.4	15.57 ± 7.37	17.15 ± 7.05	18.82 ± 7.45	<0.001
Shannon (score)	3.74 ± 0.53	3.07 ± 0.19	3.90 ± 0.29	4.25 ± 0.11	<0.001
F/B (ratio)	2.09 ± 0.61	2.81 ± 0.26	1.99 ± 0.33	1.46 ± 0.14	<0.001
Amylase (U/L)	86.27 ± 3.86	87.85 ± 3.77	85.43 ± 4.46	85.55 ± 2.65	<0.001
Lipase (U/L)	74.77 ± 3.86	76.35 ± 3.77	73.93 ± 4.46	74.05 ± 2.65	<0.001
Calprotectin (μg/g)	57.98 ± 19.42	78.73 ± 10.64	56.18 ± 14.90	39.06 ± 2.77	<0.001
IL-6 (pg/mL)	5.17 ± 1.74	7.31 ± 0.61	4.80 ± 0.92	3.42 ± 0.41	<0.001
CRP (mg/L)	3.41 ± 1.33	5.00 ± 0.67	3.09 ± 0.74	2.12 ± 0.29	<0.001
Butyrate (mmol/L)	6.49 ± 2.51	3.55 ± 0.71	6.65 ± 1.1	9.32 ± 0.95	<0.001
Hb (g/dL)	12.00 ± 1.36	11.87 ± 1.41	12.21 ± 1.35	11.91 ± 1.29	0.022
WBC (10^9/L)	7.19 ± 1.94	7.35 ± 1.98	6.95 ± 1.97	7.26 ± 1.85	0.092
NLR (ratio)	2.30 ± 0.93	2.32 ± 0.96	2.16 ± 0.93	2.41 ± 0.88	0.021
Platelets (10^9/L)	229.97 ± 51.86	232.44 ± 53.71	224.13 ± 51.60	233.50 ± 49.94	0.130
Albumin (g/dL)	3.82 ± 0.48	3.77 ± 0.51	3.85 ± 0.47	3.83 ± 0.46	0.161
AST (U/L)	33.16 ± 13.76	34.80 ± 14.37	32.23 ± 13.68	32.47 ± 13.12	0.111
ALT (U/L)	30.32 ± 12.66	31.90 ± 13.26	29.44 ± 12.50	29.64 ± 12.13	0.091
Creatinine (mg/dL)	1.06 ± 0.29	1.08 ± 0.3	1.01 ± 0.28	1.09 ± 0.28	0.015
BUN (mg/dL)	17.73 ± 4.68	17.93 ± 4.89	17.24 ± 4.67	18.04 ± 4.44	0.169
CEA (ng/mL)	4.49 ± 3.09	4.93 ± 3.25	4.2 ± 3.03	4.33 ± 2.96	0.037
CA19-9 (U/mL)	31.57 ± 23.99	35.39 ± 25.42	27.6 ± 23.73	31.82 ± 22.2	0.004
Energy (kcal)	1946.03 ± 242.81	1950.26 ± 249.74	1932.89 ± 241.25	1954.88 ± 237.88	0.621
Fiber (g)	18.52 ± 7.4	16.91 ± 7.37	18.49 ± 7.05	20.16 ± 7.45	<0.001
Carbs (g)	274.49 ± 19.54	275.24 ± 20.36	273.19 ± 18.8	275.05 ± 19.46	0.495
Protein (g)	61.49 ± 13.52	61.74 ± 13.83	60.83 ± 13.47	61.90 ± 13.29	0.681
Fat (g)	66.90 ± 13.75	66.93 ± 14.07	66.32 ± 13.87	67.45 ± 13.35	0.699
Systolic (mmHg)	128.12 ± 8.23	128.34 ± 8.33	127.84 ± 8.19	128.16 ± 8.19	0.820
Diastolic (mmHg)	81.85 ± 4.11	81.91 ± 4.32	81.59 ± 3.94	82.04 ± 4.07	0.511
Glucose (mg/dL)	91.24 ± 4.14	91.34 ± 4.43	91.01 ± 3.88	91.35 ± 4.09	0.637
HDL (mg/dL)	45.36 ± 6.97	45.35 ± 6.98	45.59 ± 6.92	45.14 ± 7.02	0.800
TG (mg/dL)	129.91 ± 4.88	129.77 ± 5.09	130.01 ± 4.5	129.94 ± 5.06	0.878
LDL (mg/dL)	95.07 ± 6.68	95.09 ± 6.73	94.98 ± 6.48	95.14 ± 6.85	0.970
Cholesterol (mg/dL)	186.46 ± 11.69	186.48 ± 11.84	185.94 ± 11.38	186.96 ± 11.87	0.672

Table displays the means, standard deviations, and *p*-values for various health metrics across three groups. *p*-values less than 0.05 indicate statistically significant differences between groups for the respective variables. Data are presented as mean ± standard deviation. *p*-values were calculated using ANOVA or Kruskal-Wallis tests where appropriate. A *p*-value < 0.05 was considered statistically significant. MIND, Mediterranean-DASH Intervention for Neurodegenerative Delay; DI-GM, Dietary Inflammatory Index—Gut Microbiota; HADS-D, Hospital Anxiety and Depression Scale—Depression Subscale; HADS-A, Hospital Anxiety and Depression Scale—Anxiety Subscale; PSQI, Pittsburgh Sleep Quality Index; FACT-C, Functional Assessment of Cancer Therapy—Colon cancer; BDNF, Brain-Derived Neurotrophic Factor; F/B ratio, Firmicutes to Bacteroidetes ratio; IL-6, Interleukin-6; CRP, C-reactive protein; WBC, White Blood Cell count; NLR, Neutrophil-to-Lymphocyte Ratio; AST/ALT, Aspartate Aminotransferase/Alanine Aminotransferase; BUN, Blood Urea Nitrogen; CEA, Carcinoembryonic Antigen; CA19-9, Cancer Antigen 19-9; HDL/LDL/TG, High-Density Lipoprotein/Low-Density Lipoprotein/ Triglycerides.

### Associations of MIND and DI-GM scores with psychological, inflammatory, and gut microbiota biomarkers

Multivariate linear regression analyses (Tables 3, 4) revealed that higher MIND and DI-GM scores were significantly associated with better psychological health, reduced systemic inflammation, and beneficial gut microbiota profiles. In fully adjusted models (Model 2), each one-unit increase in the MIND score correlated with lower depression (HADS-D *β* = −1.23, 95% CI: −1.36 to −1.10) and anxiety (HADS-A *β* = −1.23, 95% CI: −1.36 to −1.10) scores, better sleep quality (PSQI β = −0.62, 95% CI: −0.69 to −0.55), decreased inflammatory markers CRP (*β* = −0.56, 95% CI: −0.61 to −0.52) and IL-6 (*β* = −0.79, 95% CI: −0.84 to −0.74). The MIND score was also inversely associated with gut microbiota imbalance, as indicated by a lower F/B ratio (*β* = −0.24, 95% CI: −0.26 to −0.21), and reduced fecal calprotectin levels (*β* = −7.97, 95% CI: −9.08 to −6.87), suggesting attenuated intestinal inflammation. Furthermore, higher MIND scores were associated with better quality of life (FACT-C *β* = 4.42, 95% CI: 4.01 to 4.84), lower CA19-9 levels (*β* = −2.67, 95% CI: −4.21 to −1.12), and modest increases in serum BDNF (*β* = 0.24, 95% CI: 0.77 to 1.22), though the latter showed wide confidence intervals. Notably, CEA was inversely associated with MIND score (*β* = −0.32, 95% CI: −0.47 to −0.17), indicating a potentially complex relationship with tumor markers.

**Table 3:** Linear regression analysis of MIND score with psychological, inflammatory, and gut microbiota-related biomarkers in colon cancer patients.

Dependent variables	Crude	Model 1	Model 2
	β (95%CI)	β (95%CI)	β (95%CI)
HADS-D	−1.24 (−1.28, −1.20)	−1.21 (−1.34, −1.08)	−1.23 (−1.36, −1.10)
HADS-A	−1.24 (−1.28, −1.20)	−1.21 (−1.34, −1.08)	−1.23 (−1.36, −1.10)
PSQI	−0.87 (−0.89, −0.85)	−0.61 (−0.68, −0.54)	−0.62 (−0.69, −0.55)
CRP (mg/L)	−0.74 (−0.76, −0.72)	−0.56 (−0.61, −0.52)	−0.56 (−0.61, −0.52)
IL-6	−0.98 (−0.99, −0.96)	−0.79 (−0.84, −0.74)	−0.79 (−0.84, −0.74)
F/B	−0.12 (−0.15, −0.09)	−0.23 (−0.26, −0.21)	−0.24 (−0.26, −0.21)
CA19-9	−1.16 (−2.24, −0.08)	−2.48 (−4.01, −0.95)	−2.67 (−4.21, −1.12)
CEA	−0.18 (−0.32, −0.04)	−0.31 (−0.46, −0.16)	−0.32 (−0.47, −0.17)
FACT-C	5.56 (5.41, 5.70)	4.34 (3.92, 4.77)	4.42 (4.01, 4.84)
Calprotectin (μg/mL)	−10.12 (−10.50, −9.75)	−7.91 (−9.01, −6.81)	−7.97 (−9.08, −6.87)
BDNF (ng/mL)	0.83 (0.5, 1.1)	0.24 (0.77, 1.2)	0.24 (0.77, 1.22)

Crude β represents the unadjusted coefficients from the regression model. β-coefficients represent the change in the dependent biomarker per 1-unit increase in the MIND score. Model 1 includes covariates such as Age, Sex, Tumor Stage, Radiation, and Weight Loss over 6 months. Model 2 includes additional covariates such as Energy and Fiber in addition to those in Model 1.

**Table 4:** Linear regression analysis of DI-GM Score with psychological, inflammatory, and gut microbiota-related biomarkers in colon cancer patients.

Dependent variables	Crude β (95% CI)	Model 1 β (95% CI)	Model 2 β (95% CI)
HADS-D	−1.34 (−1.39, −1.29)	−1.35 (−1.51, −1.18)	−1.36 (−1.53, −1.20)
HADS-A	−1.33 (−1.39, −1.3)	−1.31 (−1.51, −1.2)	−1.35 (−1.53, −1.22)
PSQI	−0.94 (−0.97, −0.92)	−0.65 (−0.73, −0.56)	−0.65 (−0.74, −0.56)
CRP (mg/L)	−0.81 (−0.83, −0.79)	−0.66 (−0.72, −0.61)	−0.66 (−0.72, −0.61)
IL-6	−1.08 (−1.10, −1.06)	−0.95 (−1.00, −0.90)	−0.94 (−1.00, −0.89)
F/B	−0.37 (−0.38, −0.36)	−0.30 (−0.33, −0.27)	−0.30 (−0.33, −0.27)
CA19-9	−1.13 (−2.32, 0.06)	−2.95 (−4.76, −1.13)	−3.11 (−4.93, 1.29)
CEA	−0.17 (−0.32, −0.02)	−0.36 (−0.54, 0.19)	−0.38 (−0.55,-0.20)
FACT-C	6.07 (5.91, 6.23)	4.80 (4.26, 5.33)	4.85 (4.32, 5.38)
Calprotectin (μg/mL)	−11.06 (−11.48, −10.65)	−8.39 (−9.75, −7.02)	−8.40 (−9.78, −7.03)
BDNF (ng/mL)	0.89 (0.54, 1.2)	0.44 (0.77, 1.6)	0.44 (0.77, 1.6)

Crude β represents the unadjusted coefficients from the regression model. β-coefficients represent the change in the dependent biomarker per 1-unit increase in the DI-GM score. Model 1 includes covariates such as Age, Sex, Tumor Stage, Radiation, and Weight Loss over 6 months. Model 2 includes additional covariates such as Energy and Fiber in addition to those in Model 1.

Similarly, higher DI-GM scores were inversely associated with depression (HADS-D *β* = −1.36, 95% CI: −1.53 to −1.20), anxiety (HADS-A β = −1.35, 95% CI: −1.53 to −1.22), and sleep disturbances (PSQI *β* = −0.65, 95% CI: −0.74 to −0.56). Inflammatory biomarkers including CRP (*β* = −0.66, 95% CI: −0.72 to −0.61), IL-6 (*β* = −0.94, 95% CI: −1.00 to −0.89), and fecal calprotectin (*β* = −8.40, 95% CI: −9.78 to −7.03) also declined with increasing DI-GM scores. The inverse association with the F/B ratio was stronger for DI-GM (β = −0.30, 95% CI: −0.33 to −0.27) than for MIND, indicating potentially greater modulation of gut microbial balance. Higher DI-GM scores were linked with enhanced quality of life (FACT-C β = 4.85, 95% CI: 4.32 to 5.38), lower levels of CA19-9 (β = −3.11, 95% CI: −4.93 to −1.29), and modest increases in serum BDNF (β = 0.44, 95% CI: 0.77 to 1.60), while CEA was also reduced (β = −0.38, 95% CI: −0.55 to −0.20).

Overall, both MIND and DI-GM dietary scores were significantly associated with better psychological well-being, lower systemic and intestinal inflammation, and more favorable gut microbiota profiles, with the DI-GM score showing slightly stronger associations—particularly with inflammatory biomarkers and microbial imbalance—compared to the MIND score.

### Mediation analysis of the F/B ratio in DI-GM effects on psychological and inflammatory outcomes

Mediation analyses (Table 5) evaluated whether the F/B ratio mediated associations between DI-GM scores and health outcomes. DI-GM was associated with significant direct effects on depression, anxiety, inflammation, sleep quality, and quality of life. The F/B ratio partially mediated the association between DI-GM and depression (indirect effect = −0.013, 95% CI: −0.34 to −0.03) and anxiety (indirect effect = −0.07, 95% CI: −0.13 to −0.02), suggesting gut microbiota composition partly underlies the beneficial effects of DI-GM on psychological symptoms. For other outcomes (CRP, PSQI, FACT-C, calprotectin, IL-6), indirect effects were non-significant, indicating additional pathways beyond F/B ratio contribute to DI-GM’s health benefits.

**Table 5:** Mediation analysis examining the role of F/B ratio in the association between DI-GM score and health outcomes in colon cancer patients.

Outcome variables	Path	Effect type	Estimate (95% CI)
HADS-D	DI-GM → HADS-D	Direct effect	−1.34 (−1.39, −1.29)
	DI-GM → F/B ratio → HADS-D	Indirect effect	−0.013 (−0.34, −0.03)
		Total effect	−1.35 (−1.39, −1.30)
HADS-A	DI-GM → HADS-A	Direct effect	−2.51 (−2.80, −2.33)
	DI-GM → F/B ratio → HADS-A	Indirect effect	−0.07 (−0.13, −0.02)
		Total effect	−2.58 (−2.73, −2.42)
CRP (mg/L)	DI-GM → CRP	Direct effect	−0.81 (−0.83, −0.79)
	DI-GM → F/B ratio → CRP	Indirect effect	−0.01 (−0.09, 0.17)
		Total effect	−0.81 (−0.83, −0.79)
PSQI	DI-GM → PSQI	Direct effect	−0.94 (−0.97, −0.92)
	DI-GM → F/B ratio → PSQI	Indirect effect	−0.26 (−0.76, 0.22)
		Total effect	−0.97 (−1.00, −0.91)
FACT-C	DI-GM → FACT-C	Direct effect	6.07 (5.91, 6.23)
	DI-GM → F/B ratio → FACT-C	Indirect effect	0.04 (−0.02, 0.10)
		Total effect	6.11 (5.95, 6.27)
Calprotectin	DI-GM → Calprotectin	Direct effect	−11.06 (−11.48, −10.65)
	DI-GM → F/B ratio → Calprotectin	Indirect effect	−0.50 (−1.00, 0.00)
		Total effect	−11.06 (−11.48, −10.65)
IL-6	DI-GM → IL-6	Direct effect	−1.08 (−1.10, −1.06)
	DI-GM → F/B ratio → IL-6	Indirect effect	−0.05 (−0.10, 0.00)
		Total effect	−1.08 (−1.10, −1.06)

The indirect effect was calculated as the product of the coefficient from DI-GM → F/B ratio and F/B ratio → outcome. All models were fitted using maximum likelihood estimation. Confidence intervals (95%) not including zero indicate statistically significant associations at *p* < 0.05. Negative estimates reflect a decrease in the outcome variable with an increase in DI-GM score; positive estimates reflect an increase.

## Discussion

This cross-sectional study examined the associations of two dietary indices—the MIND score and the DI-GM—with psychological outcomes, systemic inflammation, gut microbiota composition, and tumor biomarkers in a large cohort of colon cancer patients. Our findings indicate that greater adherence to both MIND and DI-GM dietary patterns is significantly associated with lower levels of depression and anxiety, better sleep quality, enhanced quality of life, reduced inflammatory markers, favorable gut microbiota profiles, and lower tumor biomarker concentrations. Notably, higher DI-GM scores were associated with stronger inverse relationships with inflammatory markers and tumor biomarkers, suggesting that DI-GM may be a more sensitive indicator of diet-related associations with gut microbiota composition and systemic inflammation in this population.

Consistent with prior literature linking Mediterranean-style diets to mental health benefits in general and chronic disease populations (24, 25), higher MIND scores were significantly associated with lower levels of depressive and anxiety symptoms. Previous studies have indicated that dietary patterns rich in fruits, vegetables, and whole grains can lead to better mood and cognitive function, reinforcing the importance of nutrition in mental health management (26). We observed inverse associations between MIND adherence and psychological distress in colon cancer patients extend the evidence base to a cancer-specific context, where psychological comorbidities are prevalent and contribute to poor quality of life and treatment outcomes (27). Furthermore, better sleep quality and higher FACT-C scores among patients with higher MIND adherence reinforce the broader beneficial impact of this dietary pattern on multidimensional aspects of well-being.

Importantly, the DI-GM score, which specifically captures dietary components favoring gut microbial health, showed even stronger inverse associations with depression, anxiety, and inflammatory markers, including CRP, IL-6, and fecal calprotectin, compared to the MIND score. This suggests that dietary interventions targeting gut microbiota may provide significant psychological benefits, as previous studies have highlighted the role of gut health in emotional regulation. These results support the emerging concept that the gut microbiota is a critical intermediary linking diet to neuropsychological outcomes via the gut–brain axis (28). Mediation analysis revealed that the F/B ratio partially mediated the relationship between DI-GM adherence and psychological symptoms, highlighting the microbial community structure as a key mechanistic player. Although the F/B ratio did not mediate the diet-inflammation link, this finding aligns with literature suggesting that gut microbiota modulates mental health through mechanisms partially independent from systemic inflammation (29, 30). Additionally, higher adherence to these diets corresponded with increased Fecal butyrate levels increased by 22% with higher MIND adherence and by 35% with higher DI-GM adherence (both *p* < 0.001), indicating enhanced microbial fermentation activity likely contributing to anti-inflammatory and neuroprotective effects. Better digestive enzyme activities (amylase and lipase) observed with higher dietary adherence could also enhance nutrient absorption and gut barrier integrity, indirectly influencing systemic inflammation and mood. The use of the Shannon index and F/B ratio in this study, while limited compared to metagenomic approaches, was chosen due to their widespread application in assessing gut microbiota diversity and balance in relation to psychological and inflammatory outcomes (13, 31). The Shannon index provides a robust measure of alpha diversity, reflecting species richness and evenness, which are associated with gut health and mental well-being (13). The F/B ratio, despite being a debated marker due to its variability and lack of specificity, was included as it has been linked to inflammation and psychological distress in prior studies (4, 6). Future studies using shotgun metagenomics could provide deeper insights into microbial taxa and functional pathways.

Our observation that higher MIND and DI-GM adherence correlated with increased microbial diversity (Shannon index) and elevated fecal butyrate levels indicates that these dietary patterns foster a gut environment conducive to beneficial short-chain fatty acid (SCFA) production, which has recognized anti-inflammatory and neuroactive properties (32). Research has shown that increased microbial diversity is associated with better mental health outcomes, further emphasizing the significance of a balanced diet (13). The rise in fecal butyrate parallels previous findings demonstrating SCFAs’ capacity to maintain intestinal barrier integrity and modulate neuroimmune pathways, potentially mitigating cancer-related psychological distress (33).

The significant inverse associations between DI-GM and tumor biomarkers CA19-9 are particularly noteworthy, suggesting that dietary modulation of gut microbiota may influence tumor biology or reflect systemic tumor burden. Prior studies have suggested that dietary components can directly affect tumor growth and progression, highlighting the potential for diet to serve as a therapeutic adjunct in cancer treatment (34, 35). While causality cannot be established in this cross-sectional study, these findings echo accumulating evidence linking diet-driven microbial changes with colon cancer tumor progression and systemic (36). Given the critical prognostic value of these tumor markers in colon cancer, our data imply a potential role for microbiota-targeted nutrition interventions in cancer management. By contrast, MIND adherence showed weaker, though still significant, inverse correlations with tumor biomarkers, underscoring the specialized focus of DI-GM on microbial health.

Several mechanisms may underlie the observed associations. Dietary patterns rich in fiber, polyphenols, fermented foods, and healthy fats provide substrates for beneficial microbes, promoting microbial diversity and metabolite production. SCFAs such as butyrate can cross the gut barrier and influence central nervous system function via modulation of inflammation, neurotrophic factors, and neurotransmitter synthesis (31, 37). Moreover, reduced systemic inflammation reflected by lower IL-6 and CRP levels may decrease neuroinflammation and improve mood regulation, as chronic inflammation is implicated in depression pathogenesis (38).

Our study has important clinical implications. The strong associations of DI-GM, a gut microbiota-focused dietary index, with psychological and tumor biomarkers suggest that dietary interventions designed to enhance microbiota health could serve as adjunctive strategies to improve mental health and possibly cancer outcomes. These findings advocate for incorporating dietary counseling emphasizing microbiota-supportive foods into comprehensive care plans for colon cancer patients.

Strengths of this study include the large sample size, adjustment for multiple potential confounders, and the comprehensive assessment of diet, psychological status, inflammatory and tumor biomarkers, and gut microbiota diversity and composition. However, several limitations should be noted. As with all cross-sectional studies, reverse causation cannot be excluded; individuals with better mental health may be more likely to adhere to healthier dietary patterns. Socioeconomic factors, such as education, income, and access to nutritious foods, may also influence both diet adherence and mental health, potentially confounding observed associations. The FFQ’s 12-month recall period, administered immediately after diagnosis, may have been affected by dietary changes made in response to the cancer diagnosis. While such retrospective assessment is standard in nutritional research, it remains susceptible to recall bias and potential misclassification of habitual intake. The use of 16S rRNA sequencing limits species-level resolution and functional inference. Moreover, data on proton pump inhibitor (PPI) use and supplemental postbiotics were not collected, representing potential unmeasured confounders for microbiota-related outcomes. Additionally, the large number of statistical analyses performed increases the risk of Type I errors, which may affect the reliability of some associations. Future longitudinal and intervention studies incorporating metagenomic and metabolomic approaches are warranted to clarify causal pathways and mechanistic links.

## Conclusion

This study shows that greater adherence to MIND and DI-GM dietary patterns is associated with better mental health, lower inflammation, enhanced gut microbiota diversity, and favorable tumor biomarker levels in colon cancer patients. The DI-GM index, focused on gut microbiota health, was particularly strongly associated with reduced psychological distress and systemic inflammation. These findings support the role of gut microbiota as a key intermediary linking diet to mental well-being and inflammatory markers. Integrating microbiota-targeted nutrition with conventional treatments may improve patient quality of life.

## Data Availability

The raw data supporting the conclusions of this article will be made available by the authors, without undue reservation.
