# Correction: Associations of MIND and DI-GM dietary scores with depression, anxiety, and gut microbiota in patients with colon cancer: a cross-sectional study

**DOI:** 10.3389/fnut.2025.1731447

**Published:** 2025-11-19

**Authors:** Yaqin Meng, Jing Tian, Xiu Xiu Li, Zhou Xu

**Affiliations:** 1 Department of Psychiatry, The First Hospital of Shanxi Medical University, Taiyuan, Shanxi, China; 2 Department of Gastroenterology, Shanxi Province Cancer Hospital/Shanxi Hospital Affiliated to Cancer Hospital, Chinese Academy of Medical Sciences/Cancer Hospital Affiliated to Shanxi Medical University, Taiyuan, Shanxi, China

**Keywords:** diet, Mediterranean, biomarkers, depression, gut microbiota, colon neoplasms

The **Ethics Statement** was incorrect as published. The Ethics Statement was written as “This study was approved by the Medical Research Ethics Committee of the First Hospital of Shanxi Medical University, (Approval No. 2024.125). Written informed consent was obtained from all participants. All procedures adhered to the principles of the Declaration of Helsinki. The studies were conducted in accordance with the local legislation and institutional requirements. Written informed consent for participation in this study was provided by the participants' legal guardians/next of kin.” The correct Ethics Statement should read as “This study was approved by the Medical Research Ethics Committee of Shanxi Province Cancer Hospital (Approval No. 2024-506). Written informed consent was obtained from all participants. All procedures adhered to the principles of the Declaration of Helsinki. The studies were conducted in accordance with the local legislation and institutional requirements. Written informed consent for participation in this study was provided by the participants' legal guardians/next of kin.”

In the **Methods**, *Ethical considerations*, the approval number was erroneously given as “(Approval No. 2024.125).” The correct approval number should read as, “(Approval No. 2024-506).

The original version of this article has been updated.

